# T Cells from Programmed Death-1 Deficient Mice Respond Poorly to *Mycobacterium tuberculosis* Infection

**DOI:** 10.1371/journal.pone.0019864

**Published:** 2011-05-12

**Authors:** Sultan Tousif, Yogesh Singh, Durbaka Vijaya Raghava Prasad, Pawan Sharma, Luc Van Kaer, Gobardhan Das

**Affiliations:** 1 Immunology Group, International Centre for Genetic Engineering and Biotechnology, New Delhi, India; 2 MicroRNA and Lymphocyte Development Research Group, Department of Veterinary Basic Sciences, Royal Veterinary College, London, United Kingdom; 3 Department of Microbiology, Yogi Vamana University, Kadapa, Andhra Pradesh, India; 4 Department of Microbiology and Immunology, Vanderbilt University, School of Medicine, Nashville, Tennessee, United States of America; University of Pittsburgh, United States of America

## Abstract

**Background:**

Programmed Death-1 (PD-1; CD279) receptor molecule is widely believed to be a negative regulator predominantly expressed by exhausted/activated mouse T cells. Upon interaction with its ligands, PD-L1 and PD-L2, PD-1 inhibits activation of T cells and cytokine production, which has been documented in various viral and fungal infections as well as in vitro studies. Therefore, inhibition of T cell responses by PD-1 resulted in disease resistance in a variety of mouse infection models studied heretofore.

**Methodology/Principal Findings:**

Here, we report that PD-1 deficient (PD-1^−/−^) mice infected with *Mycobacterium tuberculosis* (*M*. *tb*) H37Rv by the aerosol route have increased susceptibility as compared with their wild type littermates. Surprisingly, *M. tb* antigen-specific T cell proliferation was dramatically reduced in PD-1 deficient animals compared with wild-type littermates, and this was due to increased numbers of regulatory T cells (Tregs) and recruitment of mesenchymal stem cells. Furthermore, PD-1^−/−^ mice exhibited decreases in the autophagy-induced LC3-B marker protein in macrophages.

**Conclusions/Significance:**

Our findings suggest that PD-1 does not play an inhibitory role during *M. tb* infection and instead promotes mycobacterial clearance in mice.

## Introduction

*Mycobacterium tuberculosis* (*M. tb*) infection is a global health problem with an estimated one third of the global population latently infected, and two million deaths annually [1], [2]. Co-infection with human immunodeficiency virus (HIV) dramatically enhances the risk of mortality and poses the threat of a pandemic in this modern era [3], [4]. Cellular immune responses, which are mediated by IFN-γ, play a central role in protective immunity against *M. tb* [5], [6]. Therefore, animals that are impaired in mounting T helper (Th) 1 responses or IFN-γ signalling exhibit dramatic susceptibility to *M. tb* infection [7], [8], [9]. On the other hand, Th2 responses exacerbate *M. tb* infection [10], [11]. Likewise, regulatory T cells (Tregs) have been shown to assist disease progression by inhibiting protective immune responses [12], [13]. Recently, we have shown that *M. tb* recruits mesenchymal stem cells (MSCs) to granulomas to promote persistent infection and Treg expansion in infected mice [14]. The role of Th17 cells in *M*. *tb* is not entirely clear, however, some reports have suggested a role of IL-17 in host resistance against *M. tb* and granuloma formation [15]. Therefore, a delicate balance between Th1, Th2, Th17, and Treg cells determines the disease outcome in *M*. *tb* infections.

A relatively newly discovered co-stimulatory receptor, Programmed Death-1 (PD-1; CD279), is a member of the CD28 family of co-stimulatory molecules, expressed by activated T, B and mesenchymal stem cells [16], [17], [18], [19], and binds with its ligands programmed death ligand 1 and 2 (PD-L1 and PD-L2; B7-H1 and B7-DC) expressed on antigen-presenting cells (APCs) [16], [18], [20], [21] and other cells. Engagement of PD-1 with its ligands has been shown to induce exhaustion in T cells, and thereby to inhibit T cell responses [22]. A number of infection studies with viral and fungal pathogens reported that PD-1/PD-L interactions inhibit T and B cell proliferation [23], [24], [25], [26], [27], and inhibition of such interactions dramatically rescues T cell functions and host resistance to infection [28]. However, some studies revealed that the PD-1/PD-L2 (B7-DC) interaction can drive the proliferation of CD4^+^ and CD8^+^ T cells [29]. Unlike viral and fungal infections, *Leishmania mexicana* infection in PD-L2^−/−^ mice resulted in exacerbated disease with no Th1/Th2 cytokine skewing, whereas PD-L1^−/−^ mice exhibited reduced IL-4 production and protection against infection [30]. Human studies indicated that PD-1/PD-L interactions inhibit *M. tb* antigen-specific peripheral T cells [31].

The divergent roles of CD4^+^ T cells (IFN-γ-producing Th1 cells and IL-4- and IL-13-producing Th2 cells) in manipulating the macrophage's phagocytic activity to control *M. tb* infection by activating and repressing autophagy, respectively, have been investigated [11], [32]. Historically, it has been known that *M. tb* resides in phagosomes and survives by interfering with phagolysosome biogenesis within macrophages or dendritic cells [33], [34]. Autophagy has a diverse role in several pathologies to protect the host against infections, cancer and aging [35]. The identification of signals that regulate autophagy and genes that execute autophagy has assisted in the development of reagents to manipulate the autophagy pathway. Activation of mammalian autophagy factor LC3 during autophagy results in a non-soluble form called LC3-II (also called LC3-B or Atg8) that stably associates with the autophagosomal membrane [36]. Autophagy can be induced by rapamycin, which can result into colocalization of the mycobacterial phagosome with the autophagy factor LC3, and induction of autophagy and suppression of intracellular survival of bacteria [34], [37], [38].

Here we report that PD-1 deficient (PD-1^−/−^) animals infected with *M. tb* exhibit higher susceptibility when compared to their wild type littermates. Consistently, the number of responding activated CD4^+^ memory T cells was dramatically lower in lung and spleen of PD-1^−/−^ animals. Furthermore, production of T cell-derived cytokines was significantly higher in PD-1^−/−^ animals. In addition to this, PD-1^−/−^ mice recruited more MSCs and Tregs to the site of infection as compared with WT littermates. Furthermore, macrophages from these animals expressed lower levels of the autophagy marker LC3-B. Taken together, these observations suggest that, unlike most other infections, PD-1 plays an important role in culminating host resistance in *M. tb* infection.

## Results

### *M. tb* H37Rv augments PD-1 expression in WT mice and enhances bacterial burden in PD-1^−/−^ mice

**Figure 1:**
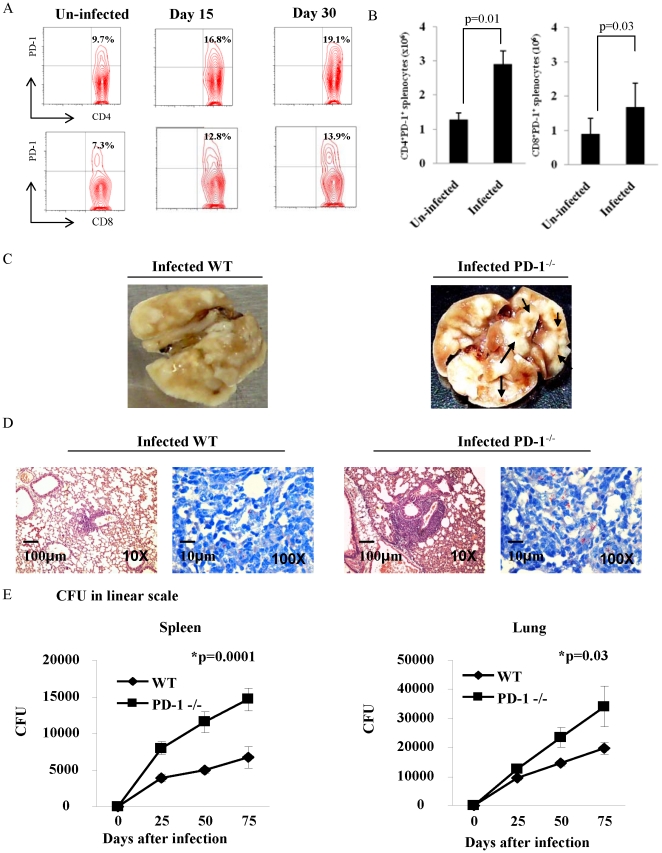
*M. tb* H37Rv augments PD-1 expression in WT mice and enhances bacterial burden in PD-1^−/−^ mice compared to WT mice. Total splenocytes from WT mice infected with H37Rv were stained with anti-CD4, anti-CD8 and anti-PD-1 using fluorescently-labelled antibodies to determine the expression of PD-1 on T cell subsets by flow cytometry. A. PD-1 expression on CD4 and CD8 cells of infected and uninfected WT mice. Cells were gated on CD4^+^ and CD8^+^ T cells and analyzed for the % of cells expressing PD-1 on the gated population. The upper dot plots (top panels) show PD-1 expression for CD4 cells, and the lower dot plots (bottom panels) show PD-1 expression for CD8 cells. Data shown here is a representative experiment of three. B. Total PD-1 costimulatory molecule expression by CD4^+^ and CD8^+^ splenocytes (mean±STDEV) in uninfected and infected WT mice. PD-1 expression is significantly higher in both CD4^+^ (p = 0.01) and CD8^+^ (p = 0.03) splenocytes, as determined by two-tailed student's t test. Data shown here is a representative experiment of three. C. Gross pathology photographs of the lungs from infected WT and PD-1^−/−^ mice. PD-1^−/−^ mice have very distinct deformed and perforated lungs after *M. tb* infection. It appears that PD-1^−/−^ mice develops cavities in the lungs, which is very unique to these mice compared to WT mice. D. Histology of the lung tissue sections after 30 days of infection in WT and PD-1^−/−^ mice stained with both Haematoxylin & Eosin and Acid Fast dyes. WT mice show very well structured granulomas compared to PD-1^−/−^ mice, whereas PD-1^−/−^ mice had very large granulomas with perforated lungs (2× larger in size as shown in the figure). PD-1^−/−^ mice developed severe lesions in the lungs after 30 days of infection with H37Rv. E. Bacterial burden is significantly higher at day 75 in PD-1^−/−^ mice compared to WT in both spleen (p = 0.0001) and lungs (p = 0.03). Data shown here is a representative of two independent experiments. Each CFU experiment has been carried out in triplicates (3 mice per experiment).

It is well established that PD-1 is expressed on activated/exhausted T cells and inhibits T cell responses. Interestingly, the amount of PD-1 expression and its engagement with ligands (PD-L1 and PD-L2) decides the threshold for T cell inactivation and the amount of cytokine production [39], [40], [41]. A study with human subjects indicated that substantial numbers of peripheral T cells express PD-1 in tuberculosis (TB) patients [31], and the inhibition of PD-1 resulted in higher *M. tb* antigen-specific proliferation *in vitro.* Therefore, we examined the expression of PD-1 on T cells from *M*. *tb* infected wild type C57BL/6 mice. It was found that both CD4^+^ and CD8^+^ T cells significantly upregulated expression of PD-1 when compared with uninfected littermates (Fig. 1A). Absolute numbers of CD4^+^ and CD8^+^ splenocytes in infected WT mice also showed significant differences in PD-1 expression compared with control mice (Fig. 1B). Therefore, to investigate the role of PD-1 in *M. tb* infection, we infected PD-1^−/−^ mice and determined bacterial burden in different organs. Consistently, lung tissue histology revealed that PD-1^−/−^ mice had very large and immature granulomas (Fig. 1C). Furthermore, PD-1^−/−^ mice have deformed and perforated lungs (gross morphology) compared to WT after *M. tb* infections (Fig. 1D). Unexpectedly and paradoxically, infected PD-1^−/−^ mice had higher bacterial load in spleen and lungs compared to infected wild type littermates (Fig. 1E). Therefore, unlike viral and fungal infections, the absence of PD-1 promotes susceptibility to *M. tb* infection.

### *M. tb* infected PD-1^−/−^ mice exhibit dramatically lower antigen-specific immune responses

**Figure 2:**
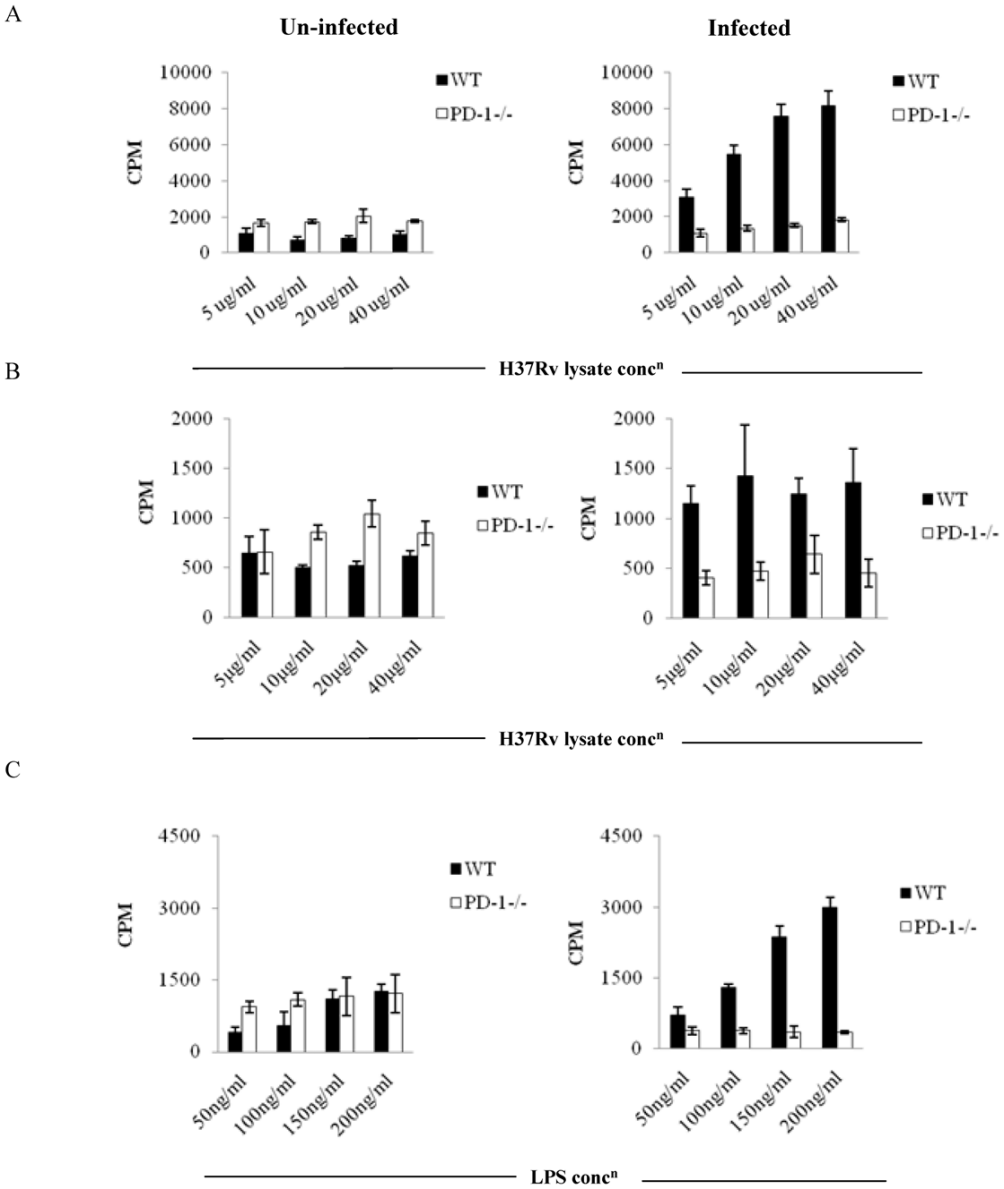
Reduced T and B cell proliferation in response to *M. tb* bacterial protein lysate (CSA) or LPS in PD-1^−/−^ mice. T or B lymphocytes were isolated from spleens and lung tissues from uninfected mice and mice infected with H37Rv. T and B cell proliferation assays were performed using tritiated thymidine. A. *In vitro* T cell proliferation of splenocytes from infected and uninfected PD-1^−/−^ mice at day 30 after infection compared to WT mice, after stimulation with *M. tb* H37Rv protein lysate. Data shown here is representative of three independent experiments with three mice in each group and represents the mean±STDEV values. B. *In vitro* proliferation of lung T cells of infected and uninfected PD-1^−/−^ mice at day 30 of infection compared to WT mice after stimulation with *M. tb* H37Rv protein lysate. Data shown here is representative of three independent experiments with three mice in each group. Each value in bar diagram is mean±STDEV of triplicate results. *C. In vitro* proliferation of splenic B cells of infected or uninfected PD-1^−/−^ mice at day 30 compared to WT mice after stimulation with varying concentrations of LPS. Data shown here is representative of three independent experiments (mean±STDEV) with three mice in each group.

**Figure 3:**
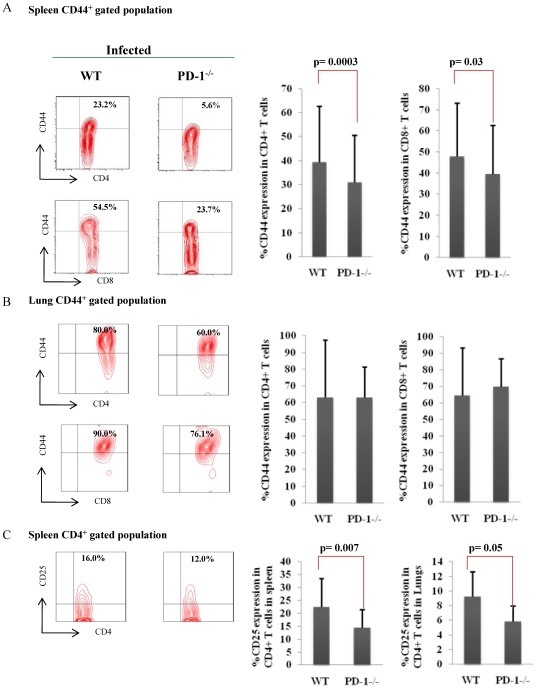
Diminished expression of CD44 by splenic CD4 and CD8 T cells. Splenocytes and lung cells from WT and PD-1^−/−^ mice infected with H37Rv were stained with anti-CD4, anti-CD8, anti-CD44 and anti-CD25 antibodies and data were acquired by flow cytometry. CD4^+^ and CD8^+^ T cells were gated against CD44. A. Reduced CD44 expression by splenic CD4 and CD8 T cells. The percentage of cells expressing CD44 among CD4^+^ and CD8^+^ T cells is shown in the bar diagram with mean±STDEV and t-test. Data shown here are representative of nine mice per group. B. CD44 expression by lung CD4 and CD8 T cells. The percentage of cells expressing CD44 among CD4^+^ and CD8^+^ T cells is shown in the bar diagram with mean±STDEV and t-test. Data shown here are representative of nine mice per group. C. CD25 expression on splenic and lung T cells. Data shown here are representative of nine mice per group. The bar diagram shows mean±STDEV value with student's t-test.

**Figure 4:**
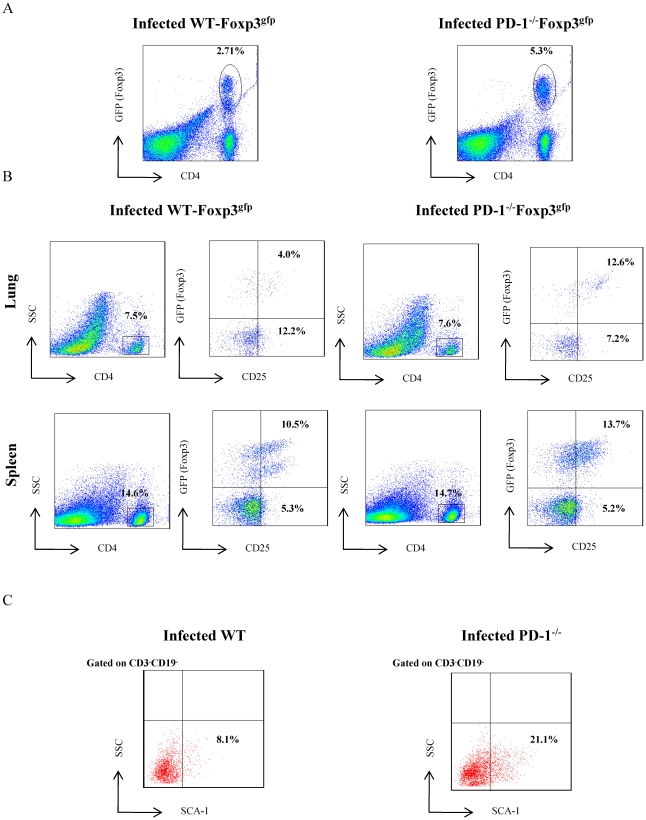
Increased numbers of Tregs in PD-1^−/−^Foxp3^gfp^ mice following *M. tb* infection. Cells isolated from both lung tissues and spleens of H37Rv-infected WT Foxp3^gfp^ and PD-1^−/−^Foxp3^gfp^ mice were stained with anti-CD4 and anti-CD25 antibodies at 30 days after infection and analyzed by flow cytometry. A. Total numbers of Tregs in infected WT (Foxp3^gfp^) and PD-1^−/−^ (Foxp3^gfp^) mice. PD-1^−/−^ mice have a two-fold increase in natural Tregs after 30 days of infection. GFP^+^ cells show the Treg cell population. B. Both lungs and spleen have increased numbers of Tregs at 30 days after infection. Total CD4^+^ T cells were gated and then expression of CD25 and Foxp3-(GFP) was analysed. C. Recruitment of mesenchymal stem cells (MSCs) in spleens of WT and PD-1^−/−^ mice. Cells were gated on CD3^−^CD19^−^SCA-1^+^ cells for analysis of MSCs. PD-1^−/−^ mice have a three-fold increase in MSCs compared to WT mice.

The higher bacterial burden in PD-1^−/−^ mice prompted us to investigate the *M. tb* antigen-specific immune responses in these animals. We examined proliferation of splenocytes and lung cells induced by *M. tb* complete soluble antigen (CSA) at day 30 after infection. Surprisingly, cells from PD-1^−/−^ animals exhibited notably reduced proliferation throughout disease progression in both lung and spleen (Fig. 2A and B). However, control uninfected PD-1^−/−^ mice did not exhibit any significant difference in T cell proliferation compared with wild type littermates in response to *M. tb* lysate protein stimulation (Fig. 2). This observation suggested that there is a defect in T cell activation in PD-1^−/−^ mice. Next, we tested whether there is any defect in B cell activation in these animals. To do so, we performed B cell proliferation assays in the presence of the B cell mitogen LPS. Indeed, we observed that B cell proliferation was also abnormal in infected PD-1^−/−^ animals compared to WT mice (Fig. 2C). An earlier time point at 15 days provided similar findings (data now shown). To provide insight into the mechanism of such unexpected findings, we examined the number of activated T cells in *M. tb* infected splenocytes. We observed that PD-1^−/−^ animals contained significantly lower numbers of CD44-positive (infected PD-1^−/−^, 30.9±19.6% [mean±STDEV] *versus* infected WT, 39.5±23.3%; n = 9, p = 0.0003) and CD25-positive (14.6±6.9% *versus* 22.5±11.0%; n = 9, p = 0.007) CD4^+^ T cells when compared with their wild type littermates in spleen (Fig. 3A, C). Similarly, in lungs, CD4^+^ T cells also showed down regulation of CD25 (5.9±2.1% versus 9.2±3.3%; n = 9, p = 0.05) expression in PD-1^−/−^ mice. The expression of CD44 was reduced in lungs of PD-1^−/−^ compared to WT animals, however no statistical significance was found (Fig. 3B). CD25 is a marker for activated T cells as well as regulatory CD4*^+^* T cells (Tregs). Therefore, we determined expression of Foxp3, a Treg specific fork-head box transcription factor, among CD25^+^ T cells in *M. tb* infected PD-1^−/−^ and wild type animals, using a Foxp3 reporter system. Tregs increased both in lungs and spleens of PD-1^−/−^ mice (Fig. 4A, B). Therefore, the differences in the proliferation were likely due to the expansion of Tregs during the progression of disease. Similarly, CD25 expression was determined on CD8^+^ T cells, but we could not detect any difference (data not shown) between WT and PD-1^−/−^ mice. Furthermore, we found that PD-1^−/−^ mice recruited more MSCs to spleens compared to WT animals (Fig. 4C). Therefore, these data suggested that the numbers of T cells responding to *M. tb* antigens are reduced due to increased numbers of Tregs and MSCs in PD-1^−/−^ mice.

### *M. tb* antigen-specific cytokine response in PD-1^−/−^ animals

**Figure 5:**
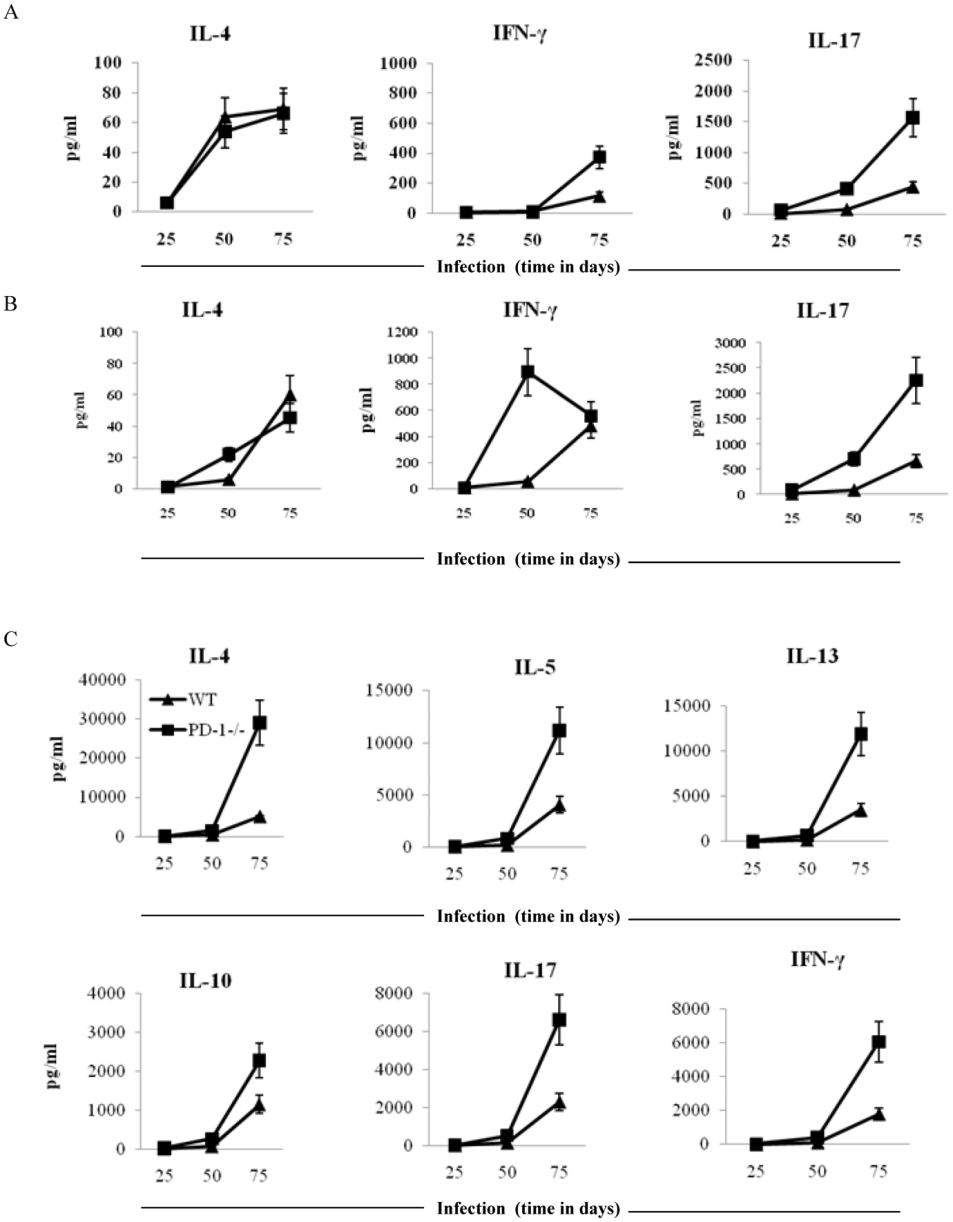
Enhanced Th1, Th2 and Th17 cytokines in PD-1^−/−^ mice infected with *M. tb*. Th1, Th2 and Th17 cytokines were assayed in the supernatant with luminex at (A) 48 hours and (B) 72 hours following activation of T lymphocytes by H37Rv protein lysate in the presence of mitomycin C-treated splenocytes. Data shown here is a representative experiment of three. (C) All cytokines in serum were present at higher amounts in PD-1^−/−^ mice compared with WT mice. Samples were collected at particular time points (days) after infection with *M. tb*. Data shown here is a representative experiment of two. At each time point three mice were used.

**Figure 6:**
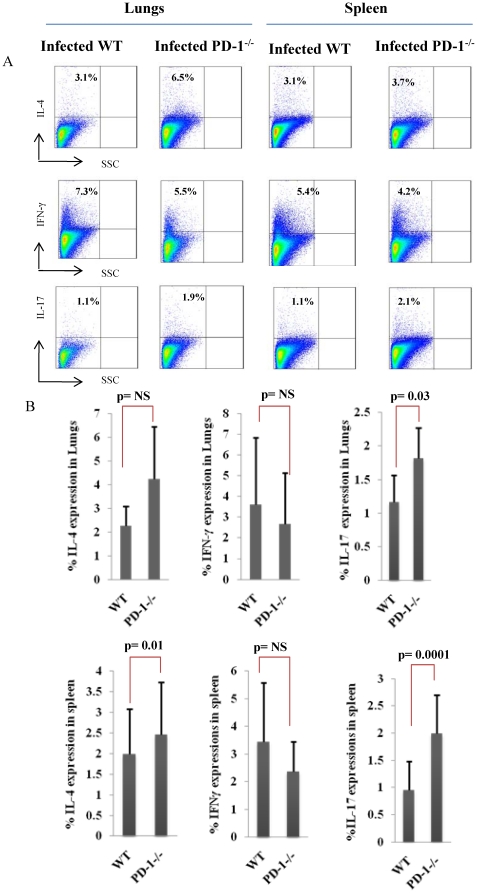
Cytokine storm in *M. tb* infected PD-1^−/−^ mice. Lung tissues and spleens were homogenised and passed through nylon wool columns. The isolated T lymphocytes were co-cultured in the presence of mitomycin C-treated syngeneic splenocytes and H37Rv protein lysate overnight in the presence of PMA and Ionomycin. The next day cells were treated with brefeldin A for four hours. Next, cells were surface stained with anti-CD4 and anti-CD8, and fixed cells were used for intracellular staining with anti-IL-4, anti-IFN-γ and anti-IL-17A antibodies. Cells were acquired on a flow cytometer. A. Total IL-4, IFN-γ and IL-17 production by lung or spleen mononuclear cells from WT and PD-1-deficient mice is shown by flow cytometry in dot plots. Data shown here is a representative experiment of three. B. Summary of the data for IL-4, IFN-γ and IL-17 production. Summary statistics show mean±STDEV and Student's t-test was applied for estimating significance between two parameters.

In the previous section, we found that PD-1^−/−^ animals induced lower *M. tb* antigen-specific T cell proliferative responses. Therefore, to examine whether antigen-specific responses exhibited a bias towards specific T helper cell subsets, we determined cytokines in the culture supernatant. We observed that PD-1^−/−^ animals produced almost equal amount of cytokines irrespective of their association with the Th1, Th2, or Th17 phenotype after twenty four hours of antigen stimulation. However, when we determined the cytokines after 48 and 72 hours of antigen stimulation, we found an increase in cytokines in PD-1^−/−^ mice (Fig. 5A, B). Similarly, serum cytokines were also higher in PD-1^−/−^ mice compared to WT mice (Fig. 5C). This observation suggested that *M. tb* antigen responding T cells in PD-1^−/−^ mice acquired an anergic phenotype, but that activation of neighbouring T cells resulted in cytokine production that overcame their anergic status. Therefore, we tested the response of spleen cells in the presence of IL-2. Supplementation of IL-2 had no effect on the proliferative responses in these animals (data not shown). Thus, we tested cytokines by intracellular cytokine staining. Surprisingly, we found that cytokines were produced by non-proliferating cells in both lungs and spleens of PD-1^−/−^ animals. Furthermore, intracellular cytokine staining revealed a significant increase in IL-17-producing cells in lungs whereas alterations in IL-4 and IFN-γ were not significant (Fig. 6A and B). In the case of spleen, there was a similar trend for IL-17 and IFN-γ, and IL-4 was found to be significantly increased as well (Fig. 6A and B). This observation suggested that *M. tb* antigen-specific T cells from PD-1^−/−^ animals may be defective in cellular proliferation. Furthermore, because of their inability to proliferate, we postulate that the capacity of these cells to consume cytokines is reduced, which results in the observed elevation of cytokines at late time points of antigen stimulation.

### *M. tb* infected PD-1^−/−^ macrophages do not undergo autophagy

**Figure 7:**
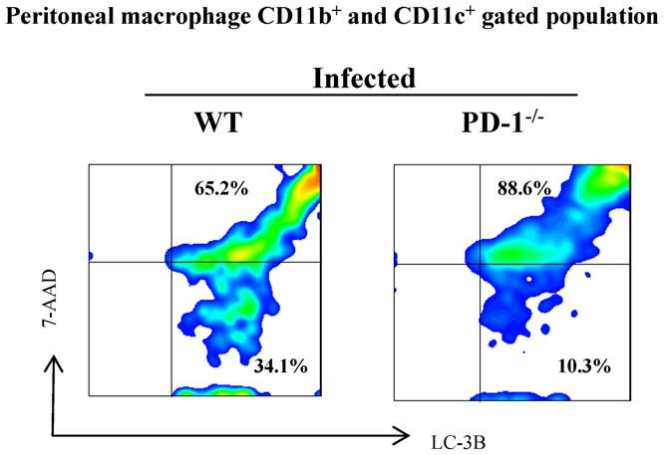
PD-1^−/−^ mice express reduced markers of autophagy after *M. tb* infection. Macrophages were isolated from the peritoneum of WT and PD-1^−/−^ mice. Peritoneal macrophages were stained with surface markers anti-CD11b and anti-CD11c and surface stained cells were intracellularly stained for the LC3-B autophagy marker. Cell viability dye 7-AAD was added before acquisition of cells by flow cytometry. Peritoneal macrophage cells were gated for CD11b and CD11c to analyze the expression of LC3-B and 7-AAD. Data shown here are representative of two experiments.

In macrophages and DCs, it has been shown that autophagy and defence against intracellular pathogens via immunity-related GTPase Irgm1 (LRG-47) has a protective role against *M. tb*, resulting in clearance of the *M. tb* organisms from macrophages [37]. We have seen very minor differences in LC3-B expression in DCs of infected lungs of WT and PD-1^−/−^ mice. However, in infected PD-1^−/−^ mice CD11b^+^ and CD11c^+^ cells had strikingly lower LC3-B expression compared with WT mice (Fig. 7). PD-1^−/−^ mice have very low autophagy and, therefore, we speculate that due to this defect these mice probably are unable to clear bacteria from the peritoneal macrophages. Nonetheless, autophagy plays a significant role in the response of PD-1^−/−^ mice to *M. tb* infection.

## Discussion

The PD-1/PD-L pathway has been extensively studied in various infection models. However, in the case of *M. tb* this pathway is still under exploration. Recently, it has been shown that PD-1^−/−^ mice have higher mycobacterial burden compared to WT infected mice and have higher serum cytokines [42], [43], and we have confirmed those findings here. When PD-1 binds to PD-L, T cells become exhausted and thereby inhibit T cell responses [22]. Various studies with viral and fungal infections reported that PD-1/PD-L interactions inhibit T and B cell proliferation [23], [24], [25], [26], [27], and inhibition of such interactions dramatically rescues T cell functions and acquired host resistance [28]. However, some studies revealed that PD-1/PD-L2 (B7-DC) interaction drives the proliferation of CD4^+^ and CD8^+^ T cells [29]. To delineate the mechanism of PD-1/PD-L action during *M. tb* infection, we have performed T and B cell proliferation assays and surprisingly, we found that PD-1^−/−^ mice after *M. tb* infection exhibit defective proliferation of lymphocytes both in lungs and spleen. Studies investigating the role of PD-1 and its ligands PD-L1 and PD-L2 in patients infected with *M. tb* suggested that blockade of this pathway enhances IFN-γ production by CD3^+^ T lymphocytes and additionaly showed that T cells also express PD-L2 [31]. Unlike these human studies, we did not observe any increase in IFN-γ production in PD-1^−/−^ mice compared to WT mice; however, we did observe reduced proliferation of T lymphocytes from PD-1^−/−^ mice. It has been shown that, in the absence of PD-1 in the mouse model of TB infection, PD-L1 binds to B7-1 molecules, which inhibits T cell proliferation and cytokine regulation [44], a finding that further support our observations in PD-1^−/−^ mice. Therefore, our data strengthen the idea of bidirectional inhibitory interactions between B7-1 and PD-L1 in the absence of PD-1 molecules.

It is well known that natural regulatory T cells help in disease progression of *M. tb* infection by inhibiting protective immune responses [12], [13]. We have also found that PD-1^−/−^Foxp3^gfp^ mice have higher numbers of natural Tregs compared to WT mice. It appears that *M. tb* recruits Treg cells to the site of infection, which is further assisted by MSCs [14]. MSCs dampen the immune response by inhibiting T cell proliferation.

A recent report also suggested that CD4^+^ T cells promote rather than inhibit the bacterial burden in PD-1-deficient mice [42]. In agreement with this report, we suggest that MSCs have an important role in the susceptibility of PD-1^−/−^ mice to *M. tb* infection. Furthermore, IFN-γ, which plays a central role in protective immunity against *M. tb* [5], [6], was found to be very similar in WT and PD-1^−/−^ mice using intracellular staining, but our PD-1^−/−^ serum data suggested that these mice have abundant amounts of accumulated IFN-γ. Therefore, PD-1^−/−^ animals, which are impaired in mounting Th1 responses or in producing IFN-γ, are susceptible to *M. tb* infection. On the other hand, Th2 responses, which exacerbate *M. tb* infections [10], [11], were found to be increased in PD-1^−/−^ animals, suggesting that these mice exhibit a severe imbalance in cytokine regulatory mechanisms due to loss of PD-1. PD-1^−/−^ mice also produce increased levels of inflammatory cytokines such IL-17, which should be protective, yet due to the concomitant increase in IL-4 production, these mice are unable to control the bacterial burden.

Our findings that macrophages from PD-1-deficient exhibit reduced autophagy suggested that these cells are unable to generate phagolysosomes to kill the engulfed bacteria [34]. Thus, overall this report suggests that PD-1-deficient mice have a defect in T cell proliferation and phagolysosomal formation. Therefore, our study provides a mechanistic link between autophagy and co-stimulatory pathways, which may be involved in higher *M. tb* bacterial growth and disease progression in PD-1-deficient animals.

In summary, our findings indicate that, in sharp contrast with other microorganisms that cause chronic infection, infection of PD-1-deficient mice with *M. tb* causes increased inflammation and bacterial load. Furthermore, we found an indirect role of reduced autophagy for the increased bacterial burden in PD-1^−/−^ mice. These findings therefore suggest a protective role of the PD-1/PD-L pathway in host immunity against *M. tb*. Manipulation of this pathway in patients infected with *M. tb* or co-infected with *M. tb* and HIV could therefore lead to unexpected outcomes.

## Methods

### Ethics Statement

All animal experiments were conducted in accordance with guidelines approved by the Institutional Animals Ethics Committee meeting held on 22^nd^ November 2007 at ICGEB (approval ID; ICGEB/IAEC/IMM-13/2007), New Delhi, India and Department of Biotechnology (DBT) guidelines, Government of India. At the relevant times after infection with *M. tb,* all mice were humanely killed by asphyxiation in carbon dioxide according to institutional and DBT regulations.

### Mice

C57BL/6 and PD-1 deficient mice (obtained from Dr. Tasuku Honjo, Yoshida-konoe, Sakyo-ku, Kyoto University, Japan, via Dr. Megan Sikes, Transplantation Biology Research Center, Harvard Medical School, Boston, MA, USA) on a C57BL/6 background were bred in our specific pathogen-free animal facility at International Centre for Genetic Engineering and Biotechnology (ICGEB), New Delhi, India. PD-1^−/−^ mice were crossed with Foxp3^gfp^ knock-in mice to obtain the PD-1^−/−^ Foxp3^gfp^ mice. Mice were housed under barrier conditions in a basic safely level III laboratory.

### Antibodies and Reagents

We used the following antibodies: anti-CD4 (clone: GK1.5)-FITC, -PerCP-Cy5 or -APC, anti-CD3 (clone: 145-2C11)-PerCP-Cy5, anti-CD19 (clone: MB19-1)-PE, SCA-1 (clone: D7)-FITC, anti-IFN-γ (clone: XMG1.2)-FITC, anti-IL-17 (clone: 17B7)-PE, anti-CD25 (clone: PC61.5)-FITC, anti-PD-1 (clone: J43)-PE antibodies (all from eBiosciences, USA), anti-CD8 (clone: 142 (53-6.7)), anti-CD44 (clone: IM7), anti-IL-4 (clone: 8D4-8) (all from BD pharmingen™, USA), anti-LC3-B (clone: Ab-2775S) (Cell signalling, USA), and IgG-Fab2 (Clone: 4412) (Cell signalling, USA).

### Flow cytometry: surface and intracellular staining

Spleens were isolated from PD-1^−/−^ and WT mice, either infected or uninfected, and macerated by frosted slides in 10% RPMI 1640 (Gibco, Invitrogen, UK) and made into a single cell suspension. Red blood cells (RBCs) were lysed with RBC cell lysis buffer, incubated at room temperature for three to five minutes and washed with 10% RPMI 1460. The cells were counted and 1×10^6^ cells were used for surface staining. For intracellular staining 1×10^6^ cells were cultured per well in 24 well plates (Nunc, USA) and activated with 50 ng/ml phorbol 12-myristate 13-acetate (PMA) and 750 ng/ml ionomycin (Sigma, USA) overnight, and 10 µg/ml brefeldin A (eBiosciences, USA) was added during the last 4 hours of culture. Cells were washed twice with PBS and stained with antibodies directed against surface markers. After staining, cells were washed again with PBS and cells were fixed with 100 µl fixation buffer (eBiociences, USA) for 30 minutes, then re-suspended in 200 µl permeabilization buffer (eBiosciences, USA) and stained with fluorescently labelled anti-cytokine antibodies. Fluorescence intensity of fluorochrome-labelled cells was measured by flow cytometry (FACS Canto™ II, BD Biosciences, USA). LC3-B staining was performed according to the manufacturer's protocol (Cell Signalling, USA) and cell viability dye (7-AAD) was added to the LC3-B stained cells 15 minutes before analyzing the cells by flow cytometry. FACS Diva was used for acquiring the cells and final data analysis was performed by Flow Jo (Tree star, USA).

### T and B cell proliferation assay

Spleens were isolated from PD-1^−/−^ and WT mice, either infected or uninfected, and macerated by frosted slides in 10% RPMI 1640 (Gibco, Invitrogen, UK) and made into a single cell suspension. Red blood cells (RBCs) were lysed with RBC cell lysis buffer and incubated at room temperature for three to five minutes and washed with 10% RPMI 1460. The total splenocytes were passed through nylon wool columns as described earlier to isolate T lymphocytes [45] and B cells were isolated using CD19^+^ magnetic beads and a B cell isolation kit using the manufacturer's instructions (Miltenyi Biotech, Germany). Similarly, lung T lymphocytes were isolated by nylon wool column method. T lymphocytes were counted and plated at 0.3×10^6^ cells/well in a 96-well plate together with equal numbers of mitomycin C (Sigma, USA) treated syngeneic splenocytes as APCs and stimulated with different concentrations of H37Rv bacterial protein lysate or *M. tb* complete soluble protein (CSA). B cells were activated with LPS (Sigma, USA) at various concentrations for B cell proliferation.Cells were cultured for 48 hours and then pulsed with tritiated thymidine (3H-TdR, 1.0 µCi per well; Amersham Biosciences, UK) before measuring incorporation of 3H-TdR by means of a cell harvester and liquid scintillation counter 16 hours later (Wallac Trilux, Perkin Elmer, UK).

### *M. tb* aerosol infection and colony forming unit (CFU) estimation

*M. tb* strain H37Rv (ATCC 27294; American Type Culture Collection, Rockville, MD) [46] infections were performed by aerosol challenge. *M. tb* strain H37Rv was grown to mid-log phase (OD600 ∼0.6) in Middlebrook 7H9 media (Difco™, USA) with 0.1% Tween 80 (Sigma, USA), 0.2% glycerol and 10% Middlebrook albumin, dextrose and catalase (ADC) enrichment medium (Difco™, USA). Bacteria were stored at −80°C in 20% glycerol stocks for further experiments. For aerosol infection, cultured stock was washed with phosphate buffer saline (PBS) twice and made into single cell suspensions. Mice were infected with ∼125 CFU of *M. tb* H37Rv using an aerosol chamber. Mice were sacrificed at various time points and organs were harvested, homogenised in 0.2 µm filtered PBS containing 0.05% Tween 80 and plated onto 7H11 Middlebrook (Difco™ USA) plates containing 10% oleic acid, albumin, dextrose and catalase (OADC) (Difco™ USA). Undiluted, ten-fold diluted and one hundred-fold diluted lung and spleen cell homogenates were plated in doublet on the above 7H11 plates and incubated at 37°C for 21 days. Colonies were counted and CFU were estimated.

### Cytokine assay

Th1, Th2 and Th17 serum cytokines were assayed by a Luminex microbead-based multiplexed (Qiagen Luminex Liquichip, USA) assay using commercially available kits according to the manufacturer's protocol (BioPlex, Bio-Rad).

### Histology staining

Lung tissues were stained with Acid Fast Bacilli (AFB) stain and Hematoxylin and Eosin (H&E) dyes as previously described [47].

### Statistical analysis

All data were analyzed by Excel 2007. In all the figures mean values were calculated with standard deviation (STDEV) until unless stated otherwise. For all statistical analyses Student's t-test was performed to compare two groups, p<0.05 was considered significant.

## References

[1] Barnes PF, Cave MD (2003). Molecular epidemiology of tuberculosis.. N Engl J Med.

[2] Dye C, Scheele S, Dolin P, Pathania V, Raviglione MC (1999). Consensus statement. Global burden of tuberculosis: estimated incidence, prevalence, and mortality by country. WHO Global Surveillance and Monitoring Project.. JAMA.

[3] Chamie G, Luetkemeyer A, Charlebois E, Havlir DV (2010). Tuberculosis as part of the natural history of HIV infection in developing countries.. Clin Infect Dis.

[4] Nunn P, Williams B, Floyd K, Dye C, Elzinga G (2005). Tuberculosis control in the era of HIV.. Nat Rev Immunol.

[5] Desvignes L, Ernst JD (2009). Interferon-gamma-responsive nonhematopoietic cells regulate the immune response to Mycobacterium tuberculosis.. Immunity.

[6] Kaufmann SH, Hengartner MO (2001). Programmed cell death: alive and well in the new millennium.. Trends Cell Biol.

[7] Baldridge MT, King KY, Boles NC, Weksberg DC, Goodell MA (2010). Quiescent haematopoietic stem cells are activated by IFN-gamma in response to chronic infection.. Nature.

[8] Cooper AM, Dalton DK, Stewart TA, Griffin JP, Russell DG (1993). Disseminated tuberculosis in interferon gamma gene-disrupted mice.. J Exp Med.

[9] Flynn JL, Chan J, Triebold KJ, Dalton DK, Stewart TA (1993). An essential role for interferon gamma in resistance to Mycobacterium tuberculosis infection.. J Exp Med.

[10] Flynn JL, Chan J (2001). Immunology of tuberculosis.. Annu Rev Immunol.

[11] Harris J, De Haro SA, Master SS, Keane J, Roberts EA (2007). T helper 2 cytokines inhibit autophagic control of intracellular Mycobacterium tuberculosis.. Immunity.

[12] Scott-Browne JP, Shafiani S, Tucker-Heard G, Ishida-Tsubota K, Fontenot JD (2007). Expansion and function of Foxp3-expressing T regulatory cells during tuberculosis.. J Exp Med.

[13] Shafiani S, Tucker-Heard G, Kariyone A, Takatsu K, Urdahl KB (2010). Pathogen-specific regulatory T cells delay the arrival of effector T cells in the lung during early tuberculosis.. J Exp Med.

[14] Raghuvanshi S, Sharma P, Singh S, Van Kaer L, Das G (2010). Mycobacterium tuberculosis evades host immunity by recruiting mesenchymal stem cells.. Proc Natl Acad Sci U S A.

[15] Okamoto Yoshida Y, Umemura M, Yahagi A, O'Brien RL, Ikuta K (2010). Essential role of IL-17A in the formation of a mycobacterial infection-induced granuloma in the lung.. J Immunol.

[16] Keir ME, Butte MJ, Freeman GJ, Sharpe AH (2008). PD-1 and its ligands in tolerance and immunity.. Annu Rev Immunol.

[17] Keir ME, Francisco LM, Sharpe AH (2007). PD-1 and its ligands in T-cell immunity.. Curr Opin Immunol.

[18] Keir ME, Latchman YE, Freeman GJ, Sharpe AH (2005). Programmed death-1 (PD-1):PD-ligand 1 interactions inhibit TCR-mediated positive selection of thymocytes.. J Immunol.

[19] Keir ME, Sharpe AH (2005). The B7/CD28 costimulatory family in autoimmunity.. Immunol Rev.

[20] Latchman Y, Wood CR, Chernova T, Chaudhary D, Borde M (2001). PD-L2 is a second ligand for PD-1 and inhibits T cell activation.. Nat Immunol.

[21] Latchman YE, Liang SC, Wu Y, Chernova T, Sobel RA (2004). PD-L1-deficient mice show that PD-L1 on T cells, antigen-presenting cells, and host tissues negatively regulates T cells.. Proc Natl Acad Sci U S A.

[22] Barber DL, Wherry EJ, Masopust D, Zhu B, Allison JP (2006). Restoring function in exhausted CD8 T cells during chronic viral infection.. Nature.

[23] Brown KE, Freeman GJ, Wherry EJ, Sharpe AH (2010). Role of PD-1 in regulating acute infections..

[24] Finnefrock AC, Tang A, Li F, Freed DC, Feng M (2009). PD-1 blockade in rhesus macaques: impact on chronic infection and prophylactic vaccination.. J Immunol.

[25] Trautmann L, Janbazian L, Chomont N, Said EA, Gimmig S (2006). Upregulation of PD-1 expression on HIV-specific CD8+ T cells leads to reversible immune dysfunction.. Nat Med.

[26] Velu V, Titanji K, Zhu B, Husain S, Pladevega A (2009). Enhancing SIV-specific immunity in vivo by PD-1 blockade.. Nature.

[27] Zhang JY, Zhang Z, Jin B, Zhang SY, Zhou CB (2008). Cutting edge: programmed death-1 up-regulation is involved in the attrition of cytomegalovirus-specific CD8+ T cells in acute self-limited hepatitis B virus infection.. J Immunol.

[28] Gotsman I, Grabie N, Dacosta R, Sukhova G, Sharpe A (2007). Proatherogenic immune responses are regulated by the PD-1/PD-L pathway in mice.. J Clin Invest.

[29] Tseng SY, Otsuji M, Gorski K, Huang X, Slansky JE (2001). B7-DC, a new dendritic cell molecule with potent costimulatory properties for T cells.. J Exp Med.

[30] Liang SC, Greenwald RJ, Latchman YE, Rosas L, Satoskar A (2006). PD-L1 and PD-L2 have distinct roles in regulating host immunity to cutaneous leishmaniasis.. Eur J Immunol.

[31] Jurado JO, Alvarez IB, Pasquinelli V, Martinez GJ, Quiroga MF (2008). Programmed death (PD)-1:PD-ligand 1/PD-ligand 2 pathway inhibits T cell effector functions during human tuberculosis.. J Immunol.

[32] Deretic V, Delgado M, Vergne I, Master S, De Haro S (2009). Autophagy in immunity against mycobacterium tuberculosis: a model system to dissect immunological roles of autophagy.. Curr Top Microbiol Immunol.

[33] Armstrong JA, Hart PD (1971). Response of cultured macrophages to Mycobacterium tuberculosis, with observations on fusion of lysosomes with phagosomes.. J Exp Med.

[34] Deretic V, Singh S, Master S, Harris J, Roberts E (2006). Mycobacterium tuberculosis inhibition of phagolysosome biogenesis and autophagy as a host defence mechanism.. Cell Microbiol.

[35] Levine B, Kroemer G (2008). Autophagy in the pathogenesis of disease.. Cell.

[36] Mizushima N, Klionsky DJ (2007). Protein turnover via autophagy: implications for metabolism.. Annu Rev Nutr.

[37] Deretic V (2006). Autophagy as an immune defense mechanism.. Curr Opin Immunol.

[38] Gutierrez MG, Master SS, Singh SB, Taylor GA, Colombo MI (2004). Autophagy is a defense mechanism inhibiting BCG and Mycobacterium tuberculosis survival in infected macrophages.. Cell.

[39] Saunders PA, Hendrycks VR, Lidinsky WA, Woods ML (2005). PD-L2:PD-1 involvement in T cell proliferation, cytokine production, and integrin-mediated adhesion.. Eur J Immunol.

[40] Sharpe AH, Wherry EJ, Ahmed R, Freeman GJ (2007). The function of programmed cell death 1 and its ligands in regulating autoimmunity and infection.. Nat Immunol.

[41] Zhong X, Bai C, Gao W, Strom TB, Rothstein TL (2004). Suppression of expression and function of negative immune regulator PD-1 by certain pattern recognition and cytokine receptor signals associated with immune system danger.. Int Immunol.

[42] Barber DL, Mayer-Barber KD, Feng CG, Sharpe AH, Sher A (2011). CD4 T cells promote rather than control tuberculosis in the absence of PD-1-mediated inhibition.. J Immunol.

[43] Lazar-Molnar E, Chen B, Sweeney KA, Wang EJ, Liu W (2010). Programmed death-1 (PD-1)-deficient mice are extraordinarily sensitive to tuberculosis.. Proc Natl Acad Sci U S A.

[44] Butte MJ, Keir ME, Phamduy TB, Sharpe AH, Freeman GJ (2007). Programmed death-1 ligand 1 interacts specifically with the B7-1 costimulatory molecule to inhibit T cell responses.. Immunity.

[45] Kokkinopoulos D, Perez S, Sotiriadou R, Stinios J, Papamichail M (1992). The use of nylon wool for the isolation of T lymphocyte subpopulations.. J Immunol Methods.

[46] Mehta R, Pearson JT, Mahajan S, Nath A, Hickey MJ (2004). Adenylylation and catalytic properties of Mycobacterium tuberculosis glutamine synthetase expressed in Escherichia coli versus mycobacteria.. J Biol Chem.

[47] Haak S, Croxford AL, Kreymborg K, Heppner FL, Pouly S (2009). IL-17A and IL-17F do not contribute vitally to autoimmune neuro-inflammation in mice.. J Clin Invest.

